# Shoulder Lump

**Published:** 1892-02

**Authors:** Wm. Stinson


### SHOULDER LUMP.

By Wm. Stinson, V. S.

On November 11th, 1891, I was called by Mr. Charles Hudson, milk-dealer, of Chelsea, Mass., to a case of so-called "Fibroid Tumor of the Shoulder." Having seen several, and dissected one out when a student, which took four weeks to heal, and this being the first case of the kind I had been called to since my graduation, I was in some doubt whether to operate at once or not, there being two "tumors," one on each shoulder, where they had been troubling him for a month or two. That on the near side was as large as a good-sized orange, and situated just above the point of the shoulder; the other was as large as a good-sized hen's egg, and a trifle lower, or just about the point of the shoulder.

Having read some time ago in your valuable journal a description of "Shoulder Lump," by Dr. Bryden, I determined to try his method. The collar in which the animal had been working, having had the padding removed, I ordered him worked for a few days with an ordinary plain leather collar, such as is used on a well shoulder. The result was, that on my return visit to him, on the 16th of November, I found that the lump on the near shouider was quite ripe and pointing, and that the one on the off shoulder was so nearly the same that I opened both, obtaining a nice, free, discharge of normal appearing pus. The parts were then dressed with a simple dressing, and the collar carefully padded, so that but little pressure came on the sores. He did a little light work for about an hour every day, and in from eight to ten days no trace of the trouble was left. The treatment proved entirely successful.

